# The Role of Smoking and Body Mass Index in Mortality Risk Assessment for Geriatric Hip Fracture Patients

**DOI:** 10.7759/cureus.26666

**Published:** 2022-07-08

**Authors:** Ariana T Meltzer-Bruhn, Garrett W Esper, Christopher G Herbosa, Abhishek Ganta, Kenneth A Egol, Sanjit R Konda

**Affiliations:** 1 Orthopedic Surgery, New York University Langone Health, New York, USA; 2 Orthopedic Surgery, Perelman School of Medicine at the University of Pennsylvania, Philadelphia, USA; 3 Orthopedic Surgery, State University of New York Upstate Medical University, Syracuse, USA; 4 Orthopedic Surgery, Washington University School of Medicine, St. Louis, USA; 5 Orthopedic Surgery, Jamaica Hospital Medical Center, New York, USA

**Keywords:** risk stratification, body mass index, smoking, geriatric, hip fracture

## Abstract

Background

Smoking, obesity, and being below a healthy body weight are known to increase all-cause mortality rates and are considered modifiable risk factors. The purpose of this study is to assess whether adding these risk factors to a validated geriatric inpatient mortality risk tool will improve the predictive capacity for hip fracture patients. We hypothesize that the predictive capacity of the Score for Trauma Triage in the Geriatric and Middle-Aged (STTGMA) tool will improve.

Methodology

Between October 2014 and August 2021, 2,421 patients >55-years-old treated for hip fractures caused by low-energy mechanisms were analyzed for demographics, injury details, hospital quality measures, and mortality. Smoking status was recorded as a current every-day smoker, former smoker, or never smoker. Smokers (current and former) were compared to non-smokers (never smokers). Body mass index (BMI) was defined as underweight (<18.5 kg/m^2^), healthy weight (18.5-24.9 kg/m^2^), overweight (25.0-24.9 kg/m^2^), or obese (>30 kg/m^2^). The baseline STTGMA tool for hip fractures (STTGMAHIP_FX_SCORE) was modified to include patients’ BMI and smoking status (STTGMA_MODIFIABLE), and new mortality risk scores were calculated. Each model’s predictive ability was compared using DeLong’s test by analyzing the area under the receiver operating curves (AUROCs). Comparative analyses were conducted on each risk quartile.

Results

A comparison of smokers versus non-smokers demonstrated that smokers experienced higher rates of inpatient (p = 0.025) and 30-day (p = 0.048) mortality, myocardial infarction (p < 0.01), acute respiratory failure (p < 0.01), and a longer length of stay (p = 0.014). Comparison among BMI cohorts demonstrated that underweight patients experienced higher rates of pneumonia (p = 0.033), decubitus ulcers (p = 0.046), and the need for an intensive care unit (ICU) (p < 0.01). AUROC comparison demonstrated that STTGMA_MODIFIABLE significantly improved the predictive capacity for inpatient mortality compared to STTGMAHIP_FX_SCORE (0.792 vs. 0.672, p = 0.0445). Quartile stratification demonstrated the highest risk cohort had a longer length of stay (p < 0.01), higher rates of inpatient (p < 0.01) and 30-day mortality (p < 0.01), and need for an ICU (p < 0.01) compared to the minimal risk cohort. Patients in the lowest risk quartile were most likely to be discharged home (p < 0.01).

Conclusions

Smoking, obesity, and being below a healthy body weight increase the risk of perioperative complications and poor outcomes. Including smoking and BMI improves the STTGMAHIP_FX_SCORE tool to predict mortality and risk stratify patient outcomes. Because smoking, obesity, and being below a healthy body weight are modifiable patient factors, providers can counsel patients and implement lifestyle changes to potentially decrease their risk of longer-term poor outcomes, especially in the setting of another fracture. For patients who are former smokers, providers can use this information to encourage continued restraint and healthy choices.

## Introduction

The worldwide population is aging. The World Health Organization (WHO) predicts that by 2030, one in six people will be 60 years old or older [1]. This trend toward an older population carries with it a higher risk of falls or accidents with subsequent orthopedic injuries. For example, the 2016 National Trauma Database found that patients older than 55 years of age comprised 42.6% of overall trauma and 57.6% of the deaths associated with these traumas [2]. Hip fractures, in particular, carry high rates of morbidity and mortality in the geriatric population [3]. Associated factors for poor outcomes in these patients include age, male gender, the presence of comorbidities, delayed time to surgery, and baseline ambulatory status [4,5]. As age, and to a certain degree, comorbidities are non-modifiable risk factors, it is important to consider factors that can be modified to lower a patient’s risk.

Body mass index (BMI) and smoking status are two such modifiable risk factors. Literature regarding the association of BMI and mortality or morbidity risk following hip fracture is divided. Despite an apparent “obesity paradox,” with obese patients having a lower risk of mortality, several studies have found contrasting results where obese, super-obese, and very underweight patients have higher rates of poor outcomes and mortality [6-9]. Similar to BMI, current research has demonstrated smoking to be associated with worse perioperative outcomes and higher rates of mortality following surgery [10-13]. Tobacco smoking is the leading cause of premature mortality that can be adjusted through behavioral changes, regardless of tobacco amount, as Qin et al. reported that even light smoking, that is, one to two cigarettes a day, can increase a patient’s all-cause mortality [14-16]. In former smokers, understanding the increased mortality risks is important to be able to provide preventative medicine and help these patients to remain smoke-free.

As hip fractures carry a significantly high rate of morbidity and mortality at baseline, it is important to consider strategies to decrease a patient’s mortality risk. Addressing and understanding modifiable risk factors is one way providers can intervene to improve outcomes. The Score for Trauma Triage in the Geriatric and Middle-Aged (STTGMA) is a validated inpatient mortality risk assessment tool for middle-aged and geriatric patients 55 and older who sustain different orthopedic trauma injuries [17]. The original STTGMA tool utilized clinical data available at the time of arrival to the emergency department (ED) to calculate a mortality risk score. Variables included in the original STTGMA tool were a patient’s age, injury details, Glasgow Coma Scale (GCS) score, and comorbidity profile as defined by the Charlson Comorbidity Index (CCI) [17]. Since STTGMA’s inception, the model has evolved to include additional variables such as a patient’s baseline ambulatory status, American Society of Anesthesiologists (ASA) score, and their coronavirus disease 2019 (COVID-19) status on hospital admission [18-20].

The purpose of this study is to determine whether the inclusion of two additional modifiable clinical variables, BMI and smoking status, would further improve the predictive capacity and risk stratification regarding inpatient mortality for geriatric and middle-aged patients treated for hip fractures. We hypothesize that the addition of these modifiable risk factors will improve predictive capacity.

## Materials and methods

This is a retrospective cohort study. An Institutional Review Board-approved trauma database was queried for all patients aged 55 and older who sustained a low-energy hip fracture (low energy defined as a fall from standing or from a height of fewer than two stairs) between October 2014 and August 2021. All patients were treated at one urban academic medical center. Fracture patterns included in our analysis were subtrochanteric, femoral neck, or intertrochanteric hip fractures [AO Foundation/Orthopaedic Trauma Association fracture classifications: 31A, 31B, 32(A-C)]. Patients were excluded if they were younger than 55 years old or had a high-energy mechanism of injury.

Each patient’s chart was reviewed for demographics that included age, BMI, gender, smoking status, baseline ambulatory status, and comorbidities using CCI. Smoking status was recorded as a current every-day smoker, former smoker, or never smoker. On a pre-study analysis, as current and former smokers were found to have no differences in complication, hospital quality, or mortality rates, these patients were grouped for analysis. Therefore, patients were considered smokers if they were current or former smokers. BMI was defined as underweight (<18.5 kg/m^2^), healthy weight (18.5-24.9 kg/m^2^), overweight (25.0-24.9 kg/m^2^), or obese (>30 kg/m^2^). Injury presentation variables collected were GCS scores and Abbreviated Injury Severity scores (AIS) for both the Head/Neck (AIS H/N) and Chest (AIS C).

Hospital quality measures collected were the length of stay (LOS) in days, the need for admission to the Intensive Care Unit (ICU), and discharge home (home was defined as either home independently or home with a health service). Mortality measures collected included inpatient and 30-day mortality. Inpatient complications recorded during each patient’s admission included sepsis/septic shock, pneumonia, deep vein thrombus/pulmonary embolism (DVT/PE), myocardial infarction (MI), acute renal failure/acute kidney injury (AKI), stroke, surgical site infection (SSI), decubitus ulcer, urinary tract infection (UTI), acute respiratory failure (ARF), anemia, and cardiac arrest.

Patients were initially grouped based on their smoking status, smokers (current and former) versus non-smokers (never smokers), and BMIs. Comparative analyses were conducted between each of these cohorts. For each patient, the baseline STTGMA score for hip fractures (STTGMAHIP_FX_SCORE) was calculated. The model was then adapted to include a patient’s BMI and smoking status (current every-day smoker, former smoker, or never smoker). A new mortality risk score, STTGMA_MODIFIABLE, was calculated for each patient. The predictive ability of each model was then compared using DeLong’s test to assess the area under the receiver operating curves (AUROCs). Then, patients were stratified into risk quartiles based on their new respective STTGMA_MODIFIABLE mortality risk scores. Comparative analyses were conducted on each risk quartile to assess the efficacy of the new BMI and smoking status factors.

The following statistical tests were used as appropriate: Mann-Whitney U tests, chi-square tests, independent-sample t-tests, and analysis of variance (ANOVA). All statistics were calculated using SPSS Version 25 (IBM Corp., Armonk, NY, USA). The significance for this study was defined with an alpha of 0.05.

## Results

In total, 2,421 patients met the inclusion criteria. Characteristics for the total cohort were as follows: 69% of patients were female, the mean age was 80.7 ± 10.2 years, mean BMI was 24.17 ± 4.94 kg/m^2^, median GCS score was 15 (interquartile range (IQR): 0), mean CCI was 1.49 ± 1.73, mean AIS Head/Neck was 0.03 ± 0.27, and mean AIS Chest was 0.02 ± 0.19. The majority of patients were White (71.71%). At baseline, most patients were community ambulators (67.91%), while 28.17% of patients were household ambulators, and 3.92% were non-ambulatory (Table 1).

**Table 1:** Demographics of the overall cohort.

Cohort demographics	Total n (%)
N	2,421
Age	80.70 ± 10.20
Body mass index	24.17 ± 4.94
Charlson Comorbidity Index	1.49 ± 1.73
Male	739 (30.52%)
Female	1,682 (69.48%)
White	1,736 (71.71%)
Black	190 (7.85%)
Hispanic	131 (5.41%)
Asian	202 (8.34%)
Other	48 (1.98%)
Unknown	114 (4.71%)
Community ambulator	1,644 (67.91%)
Household ambulator	682 (28.17%)
Non-ambulatory/Wheelchair	95 (3.92%)
Glasgow Coma Scale score	15 (interquartile range: 0)
Abbreviated Injury Score Head/Neck	0.03 ± 0.27
Abbreviated Injury Score Chest	0.02 ± 0.19

An initial comparison of the current versus former smoker cohorts demonstrated that there were no differences in complication risk, hospital quality measures, or mortality outcomes (p > 0.05 for all). Subsequently, former and current smokers were grouped for further analysis. When comparing the smoker versus non-smoker cohorts, patients who were currently smoking or had a history of smoking experienced higher rates of inpatient (2.85% vs. 1.52%, p = 0.025) and 30-day (5.60% vs. 3.88%, p = 0.048) mortality. They also had higher rates of MI (2.01% vs. 0.76%, p < 0.01) and ARF (6.98% vs. 3.39%, p < 0.01), and had a longer inpatient LOS (in days: 6.82 ± 4.83 vs. 6.28 ± 4.17, p = 0.037) (Table 2).

**Table 2:** Comparison of outcomes between smoker and non-smoker cohorts. DVT/PE = deep vein thrombosis/pulmonary embolism; MI = myocardial infarction; AKI = acute kidney injury; SSI = surgical site infection; UTI = urinary tract infection; ARF = acute respiratory failure; LOS = length of stay; ICU: intensive care unit; SD: standard deviation

Outcomes	Smoker n (%)	Non-smoker n (%)	P-value
N	946	1,445	
Sepsis/Septic shock	27 (2.85%)	26 (1.80%)	0.085
Pneumonia	53 (5.60%)	59 (4.08%)	0.084
DVT/PE	23 (2.43%)	24 (1.66%)	0.182
MI	19 (2.01%)	11 (0.76%)	<0.01
AKI	91 (9.62%)	107 (7.40%)	0.054
Stroke	5 (0.53%)	6 (0.42%)	0.686
SSI	1 (0.11%)	2 (0.14%)	0.826
Decubitus ulcer	17 (1.80%)	16 (1.11%)	0.155
UTI	64 (6.77%)	123 (8.51%)	0.124
ARF	66 (6.98%)	49 (3.39%)	<0.01
Anemia	278 (29.39%)	456 (31.56%)	0.277
Cardiac arrest	12 (1.27%)	17 (1.18%)	0.835
LOS (d, mean ± SD)	6.82 ± 4.83	6.28 ± 4.17	0.014
Need for ICU	170 (17.97%)	277 (19.17%)	0.462
Discharged home	223 (23.57%)	338 (23.39%)	0.933
Inpatient mortality	27 (2.85%)	22 (1.52%)	0.025
30-day mortality	53 (5.60%)	56 (3.88%)	0.048

Comparison among BMI cut-off cohorts demonstrated that underweight patients experienced higher rates of pneumonia (p = 0.033), decubitus ulcers (p=0.046), and need for the ICU (p < 0.01) (Table 3).

**Table 3:** Comparison of outcomes between body mass index groups. DVT/PE = deep vein thrombosis/pulmonary embolism; MI = myocardial infarction; AKI = acute kidney injury; SSI = surgical site infection; UTI = urinary tract infection; ARF = acute respiratory failure; LOS = length of stay; ICU: intensive care unit; SD: standard deviation

Outcomes	BMI <18.5	BMI 18.5-24.9	BMI 25.0-29.9	BMI 30.0+	P-Value
N	946	1,445	946	1,445	
Sepsis/Septic shock	5 (2.02%)	29 (2.44%)	13 (1.86%)	7 (2.43%)	0.854
Pneumonia	20 (8.06%)	58 (4.89%)	25 (3.58%)	11 (3.82%)	0.033
DVT/PE	5 (2.02%)	23 (1.94%)	14 (2.01%)	5 (1.74%)	0.994
MI	2 (0.81%)	18 (1.52%)	8 (1.15%)	2 (0.69%)	0.598
AKI	15 (6.05%)	93 (7.83%)	62 (8.88%)	32 (11.11%)	0.150
Stroke	1 (0.40%)	6 (0.51%)	2 (0.29%)	2 (0.69%)	0.828
SSI	1 (0.40%)	2 (0.17%)	1 (0.14%)	0 (0.00%)	0.719
Decubitus ulcer	8 (3.23%)	16 (1.35%)	7 (1.00%)	2 (0.69%)	0.046
UTI	21 (8.47%)	98 (8.26%)	51 (7.31%)	20 (6.94%)	0.801
ARF	17 (6.85%)	44 (3.71%)	39 (5.59%)	17 (5.90%)	0.071
Anemia	74 (29.84%)	386 (32.52%)	201 (28.80%)	83 (28.82%)	0.309
Cardiac arrest	2 (0.81%)	18 (1.52%)	6 (0.86%)	3 (1.04%)	0.557
LOS (d, mean ± SD)	6.81 ± 4.82	6.37 ± 4.23	6.48 ± 4.20	6.85 ± 5.42	0.265
Need for ICU	64 (25.81%)	217 (18.28%)	133 (19.05%)	40 (13.89%)	<0.01
Discharged home	58 (23.39%)	277 (23.34%)	170 (24.36%)	68 (23.61%)	0.964
Inpatient mortality	6 (2.42%)	25 (2.11%)	9 (1.29%)	10 (3.47%)	0.167
30-day mortality	14 (5.65%)	55 (4.63%)	26 (3.72%)	18 (6.25%)	0.314

**Figure 1:**
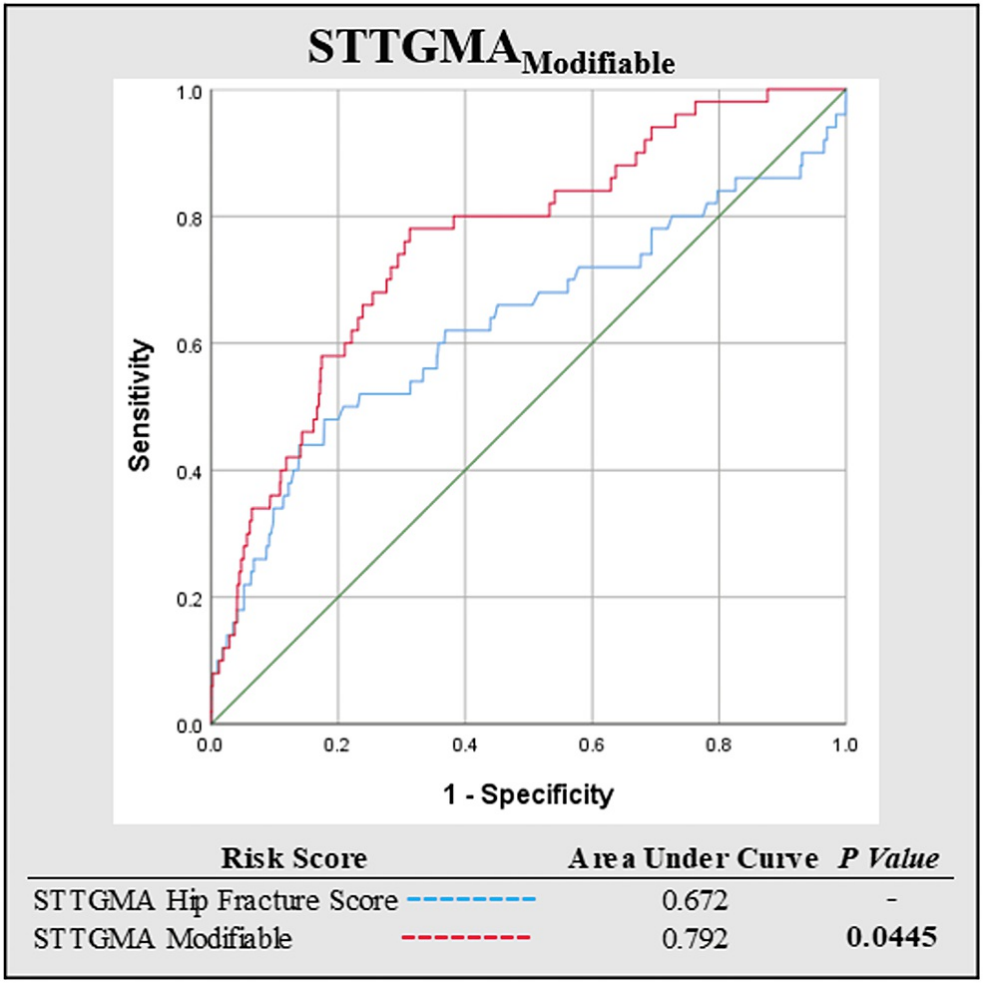
Comparison of the area under the receiver operating curve for the STTGMAHIP_FX_SCORE and STTGMA_MODIFIABLE mortality risk scores. STTGMA = Score for Trauma Triage in the Geriatric and Middle-Aged

When comparing each risk score’s respective AUROC, STTGMA_MODIFIABLE was found to improve the predictive capacity for inpatient mortality compared to STTGMAHIP_FX_SCORE (0.792 vs. 0.672, p = 0.0445) (Figure 1).

Regression weighting showed a coefficient of 0.337, with current smokers having the greatest absolute effect size (current every-day smoker = 1.011, former smoker = 0.674). While our other study findings demonstrate being below a healthy body weight increases the risk of inpatient mortality, BMI had a regression coefficient of 0.116, suggesting that a very high BMI is more strongly positively correlated with a higher risk of inpatient mortality. While this demonstrates statistically that a higher BMI positively correlates with a higher risk of inpatient mortality, our additional study findings demonstrate being below a healthy body weight similarly increases the risk of inpatient mortality.

When comparing risk quartiles for STTGMA_MODIFIABLE, multiple outcomes had significance. For mortality, patients in the highest risk quartile (STTGMA score >2.50%) experienced the highest rates of both inpatient (p < 0.01) and 30-day (p < 0.01) mortality. Patients in the highest risk cohort similarly experienced a longer inpatient LOS (p < 0.01), higher rates of sepsis (p < 0.01), pneumonia (p < 0.01), DVT/PE (p = 0.015), MI (p = 0.032), AKI (p < 0.01), ARF (p < 0.01), anemia (p < 0.01), cardiac arrest (p < 0.01), need for ICU level of care (p < 0.01), and were the least likely to be discharged home (p < 0.01) (Table 4).

**Table 4:** Comparison of outcomes between the STTGMA_MODIFIABLE risk quartiles. DVT/PE = deep vein thrombosis/pulmonary embolism; MI = myocardial infarction; AKI = acute kidney injury; SSI = surgical site infection; UTI = urinary tract infection; ARF = acute respiratory failure; LOS = length of stay; ICU: intensive care unit; SD: standard deviation; STTGMA = Score for Trauma Triage in the Geriatric and Middle-Aged

Outcomes	High risk, n (%)	Moderate risk, n (%)	Low risk, n (%)	Minimal risk, n (%)	P-value
	(100-75%)	(75-50%)	(50-25%)	(25-0%)	
STTGMA risk score	>2.50%	2.49%-1.36%	1.35%-0.79%	<0.78%	
N	606	605	605	605	
Sepsis/Septic shock	32 (5.28%)	11 (1.82%)	3 (0.50%)	8 (1.32%)	<0.01
Pneumonia	53 (8.75%)	26 (4.30%)	21 (3.47%)	14 (2.31%)	<0.01
DVT/PE	19 (3.14%)	14 (2.31%)	10 (1.65%)	4 (0.66%)	0.015
MI	13 (2.15%)	9 (1.49%)	6 (0.99%)	2 (0.33%)	0.032
AKI	87 (14.36%)	64 (10.58%)	26 (4.30%)	25 (4.13%)	<0.01
Stroke	4 (0.66%)	4 (0.66%)	0 (0.00%)	3 (0.50%)	0.269
SSI	2 (0.33%)	1 (0.17%)	0 (0.00%)	1 (0.17%)	0.572
Decubitus ulcer	12 (1.98%)	9 (1.49%)	8 (1.32%)	4 (0.66%)	0.256
UTI	61 (10.07%)	50 (8.26%)	42 (6.94%)	37 (6.12%)	0.055
ARF	53 (8.75%)	27 (4.46%)	23 (3.80%)	14 (2.31%)	<0.01
Anemia	204 (33.66%)	208 (34.38%)	169 (27.93%)	163 (26.94%)	<0.01
Cardiac arrest	18 (2.97%)	7 (1.16%)	2 (0.33%)	2 (0.33%)	<0.01
LOS (d, mean ± SD)	7.48 ± 5.26	6.60 ± 4.12	6.03 ± 4.13	5.88 ± 3.98	<0.01
Need for ICU	160 (26.40%)	112 (18.51%)	108 (17.85%)	74 (12.23%)	<0.01
Discharged home	88 (14.52%)	104 (17.19%)	148 (24.46%)	233 (38.51%)	<0.01
Inpatient mortality	34 (5.61%)	8 (1.32%)	5 (0.83%)	3 (0.50%)	<0.01
30-day mortality	64 (10.56%)	30 (4.96%)	12 (1.98%)	7 (1.16%)	<0.01

## Discussion

The purpose of this study was to assess if the addition of various modifiable risk factors, a patient’s BMI and smoking status, to a validated inpatient mortality risk assessment tool improved the model’s predictive capacity and ability to effectively triage geriatric and middle-aged patients treated for hip fracture. This study demonstrates that the addition of these modifiable risk factors provided an improved predictive model. This improved mortality risk model will help guide treatment decisions and provide valuable prognostic information to discuss expectations surrounding patients’ injuries and potential outcomes with patients and their families.

This study demonstrated that patients who are either current smokers or have a history of smoking are at a higher risk for perioperative complications and potentially worse outcomes. While the higher mortality rate cannot be linked solely to a patient’s smoking status, the higher mortality rates found in our study align with those reported in the literature [11,12]. The higher rates of MI and ARF seen in smokers can be expected as well due to the well-documented cardiovascular and pulmonary diseases found in patients secondary to smoking history [21,22]. Longer hospitalizations may also be attributed to the higher complication rates as patients in the smoker cohort required extended hospital stays to improve their health status before discharge. Similarly, these patients had worse baseline statuses prior to the injury, potentially necessitating a longer inpatient course. In addition, it is well documented in the literature that smoking delays wound healing [23-25]. For patients who required surgery as a part of their treatment for hip fracture, it is possible that they needed a longer time to heal due to the detrimental wound healing effects caused by smoking. While in our study, the rate of decubitus ulcer was higher in the smoker cohort, it was not significant. This may be due to the size of our patient cohort; given a larger patient cohort, we may have seen higher rates of decubitus ulcers. In addition, we did not capture the rate of wound infections which could also impact LOS. While the causes of the higher perioperative and mortality rates are multifactorial, smoking likely played a role. Smoking cessation has been proven to improve underlying cardiovascular and pulmonary health [26]. Providers may use this knowledge to counsel patients on the importance of both smoking cessation and/or continuing to remain smoke-free.

This study also demonstrated that patients who are underweight are at a higher risk for perioperative complications and potentially worse outcomes. Patients with a BMI of less than 18.5 kg/m^2^ were found to be at higher risk for pneumonia, decubitus ulcers, and the need for the ICU. Patients who are underweight may be malnourished and have vitamin deficiencies that impact immune function and wound healing, placing them at higher risk for skin breakdown. An international pressure ulcer prevalence survey and a study by Hyun et al. found that underweight and extremely obese patients were at higher risk for pressure ulcers [27]. Several studies have shown that the risk of infection, such as pneumonia, follows a U-shaped curve, suggesting that both underweight and obese patients are at higher risk [28,29]. While we saw a higher risk of pneumonia in the underweight cohort, it is possible that in our study, by not further splitting super-obese patients from obese patients, we did not see a higher risk of pneumonia in the higher BMI group. Additionally, while our study found that patients who are below a healthy body weight also had a higher risk of inpatient mortality, our regression showed that the higher a patient’s BMI, the higher the risk of inpatient mortality, similar to that found in the literature [8]. Obesity is associated with several comorbidities such as diabetes, heart disease, and increased risk for stroke, all health issues that have higher rates of mortality [27,30]. Patients who are underweight or obese can be identified as higher risk on arrival, allowing for timely intervention and appropriate medical management. Prior to discharge, counseling can be provided on effective nutrition plans and active lifestyle adjustments to help patients attain healthy body weight.

The inclusion of these modifiable risk factors in the STTGMA tool allowed for effective triage of high-risk patients into appropriate risk quartiles. Stratification using STTGMA_MODIFIABLE identified patients who experienced not only higher rates of inpatient mortality, 30-day mortality, and the need for the ICU but also higher rates of serious inpatient complications such as sepsis, DVT/PE, MI, and AKI. Providers may use these added clinical variables to better identify patients who may require more intensive medical management and timely intervention. This may have implications to improve outcomes and reduce hospital costs by proactively managing patients to help lower complication and mortality rates while shortening hospital admissions.

This study has several limitations. First, as a retrospective study, it is subject to the common biases associated with this study format. Second, our analysis relied upon database entries for a patient’s smoking status. Therefore, we were unable to assess a patient’s smoking status if it was not recorded in the electronic medical record (EMR). However, as only 30 patients did not have a smoking status EMR entry, the impact of this limitation may be minimal. Third, our study did not include the number of cigarettes, packs, and pack-years for each patient. As the adverse effects of tobacco smoking may have a dose-dependent relationship, our analysis could not account for this component of a patient’s smoking status. Further analysis may be done to include a weighting factor that considers smoking amount. Fourth, our study did not distinguish super-obese patients from obese patients. There may be an additional risk or protective factors associated with super-obese patients. Additional studies may also be conducted to include a cost analysis to assess the impact of different BMIs and smoking status on hospital costs. Lastly, future studies may focus on a prospective analysis comparing mortality risks overtime in a cohort that modifies its risk (i.e., by losing weight or stopping smoking) versus a cohort that does not.

## Conclusions

Smoking, obesity, and being below a healthy body weight increase the risk of perioperative complications and poor outcomes. Including smoking and BMI improves the STTGMAHIP_FX_SCORE tool to predict mortality and risk stratify patient outcomes. Because smoking, obesity, and being below a healthy body weight are modifiable patient factors, providers can counsel patients and implement lifestyle changes to potentially decrease their risk of longer-term poor outcomes, especially in the setting of another fracture. For patients who are former smokers, providers can use this information to encourage continued restraint and healthy choices.
